# Relationship of Mental Stress of Middle School Students and Campus Safety Atmosphere with Psychosocial Safety Behaviors

**Published:** 2020-04

**Authors:** Jinchang YANG, Wei YAN

**Affiliations:** 1. School of Safety Science and Emergency Management, Wuhan University of Technology, Wuhan, Hubei, China; 2. Department of Finance, Guangxi Financial Vocational College, Nanning, Guangxi, China

**Keywords:** Mental stress, Psychology, Behavior

## Abstract

**Background:**

Incidents of violence, such as school bullying, are mainly caused by excessive mental stress of students, which will also lead to all kinds of psychologically unsafe behaviors. The emotion regulation ability of students and the safety atmosphere level of campus will be considerably conducive to relieving the mental stress of students. This study aims to analyze the relationships of mental stress and regulatory emotional self-efficacy (RES) among students and campus safety atmosphere with their psychosocial safety behaviors (PSB).

**Methods:**

A total of 120 class teachers and 365 students from three junior high schools in Henan Province, China were selected as the study objects in 2019. Then, middle school students and RES, campus safety atmosphere, and PSB scales were assessed through a mental stress scale.

**Results:**

Mental stress (*r*=–0.8) of middle school students and campus safety atmosphere (*r*=0.86) had a significant negative and positive influence on their PSB, respectively. RES played a mediating role in the negative correlation between the mental stress of middle school students and their PSB (*r*=–0.57). Campus safety atmosphere could moderate the relationship between mental stress and RES (*r*=0.12). Campus safety atmosphere could moderate the mediating effect on the relationship between mental stress of middle school students and their PSB.

**Conclusion:**

Mental stress of middle school students will give rise to the occurrence of their psychologically unsafe behaviors. The improvement of the campus safety atmosphere level can effectively mitigate the mental stress of students to reduce the occurrence of unsafe behaviors.

## Introduction

Incidents of school violence and bullying happen frequently in recent years through all phases of studying from primary, middle, and high school to university. These incidents will not only cause bodily harm but also induce a series of psychological health problems. Among various phases of study during which incidents of school violence and bullying take place, middle school students highly suffer from school bullying (1).

Then what are the causes of frequent unsafe incidents such as school violence and bullying? Mental stress and psychological problems are the primary causes of unsafe behaviors (2, 3). Campus violence and bullying always was a research hotspot in the field of campus safety (4, 5). Therefore, how to mitigate the mental stress of students and reduce the occurrence of unsafe accidents, such as school violence and bullying, has become one of the problems needing urgent solutions in campus safety today.

Research involving school violence of adolescents indicates that different gender expressions present a certain linear relation with bullying behaviors. Thus, schemes preventing incidents of school violence should include gender diversity-oriented education (6). Attention deficit hyperactivity disorder is not only the primary cause for school bullying; students with this disorder are the main victims of school bullying, and hyperactivity patients are more likely to become victims of bullying incidents than ordinary students (7). In addition, the relationship between mental regulation of adolescents and network bullying behaviors is studied.

In comparison with those who are not bullied, those who are severely bullied online usually have heavy perceptive pressure, aloneness, and depression with a lack of self-confidence including low life satisfaction (2). Nowadays, most of studies just investigated the current situation of mental stress and psychological problems (2,3). In addition, studies exploring the relationships of mental stress with unsafe behaviors, such as violence and bullying are limited. Bronkhorst called safety behavior related to psychological health psychosocial safety behavior (PSB) and put forward that PSB was an important constituent part of human safety behaviors (8).

Regulatory emotional self-efficacy (RES) of adolescents can effectively mitigate the negative correlation between mental stress and depression (9–11). Moreover, RES can reduce unsafe behaviors, such as school violence, so as to increase safety behaviors. In addition, safety atmosphere plays a significant role in improving safety behaviors of members in an organization. A high safety atmosphere level can reduce the occurrence rate of safety accidents (10), and the influence of mental stress on RES and PSB may rely on campus safety atmosphere level. However, most studies have just discussed the influence of enterprise safety atmosphere on safety behaviors of employees, and few have involved campus safety atmosphere (11).

Therefore, from the perspective of psychological safety of middle school students, the present study aimed to integrate studies regarding mental stress and PSB of middle school students. The objective was to discuss the influence mechanism of their mental stress on their PSB, the mediating role played by RES, and the regulating effect of campus safety atmosphere. The present study also expects to provide a theoretical basis and scientific guidance for effectively relieving mental stress of middle school students, reducing school violence and bullying, and improving campus safety management level.

## Methods

### Description of Variables and Empirical Model

**Fig. 1:**
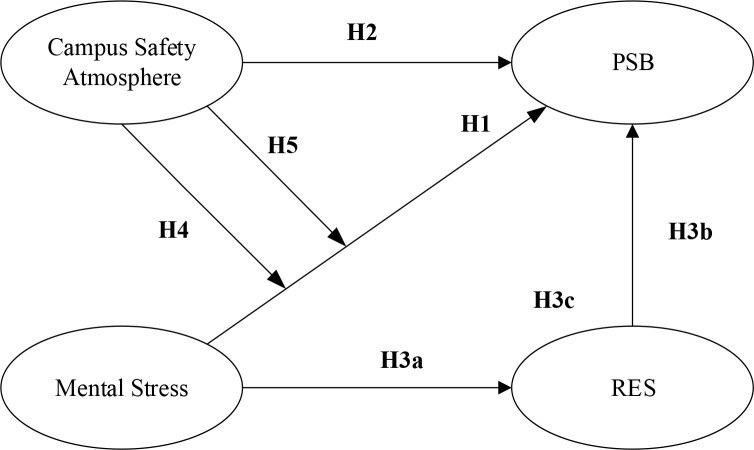
Theoretical model

Our empirical model was based on a study (8), extended by others (12–15). The latter studies examined the relationship between psychological stress, campus safety atmosphere, emotionally regulated self-efficacy, and unsafe behavior. We extended framework of (12–15) by including the interaction of all factors. The study focused on three junior high schools in Henan, China that considerably take place bully events (Fig. 1).

Where PSB denotes the psychosocial safety behaviors. PSB of middle school students is defined as campus activities that they actively participate in and proactively carry out to maintain their own psychological safety (8). In today’s society, middle school students are facing pressures from various aspects, such as family, school work, interpersonal relationship, and school group. These pressures will generate an enormous negative effect on their psychological health (12). In addition, if they bear high pressure, then they may generate unsafe behaviors, such as bullying, violence, and suicide (13). Hence, starting from the angles of psychological health and safety, we believe that mental stress of middle school students presents a significant negative correlation with their PSB.

Campus safety atmosphere refers to the perception and evaluation made by students for the importance degree attached by the school to their safety and health (11). Safety investment, management, and atmosphere of a school are significantly associated with unsafe behaviors of students (14). Schools should pay high attention to campus safety atmosphere. Therefore, campus safety atmosphere may present a significant positive correlation with PSB of middle school students.

RES denotes the regulatory emotional self-efficacy. RES means the individual confidence level in their emotion regulation ability, including self-efficacy regulating positive and negative emotions. In addition, a negative emotion includes self-efficacy regulating depression/pain and anger/rage. RES reflects a kind of psychological confidence state in self-decompression and self-regulation of individuals in their life, study, and work and plays a significant role in individual PSB. RES varies from individual to individual, and emotion regulation abilities of the same individual will be very different under different mental states. When a student is faced with high mental stress, his/her RES will be weakened (15). Moreover, if the confidence level of students in their emotion regulation ability is high, then they feel happy in a happy event. When encountering depressing or irritating events, they will effectively regulate their depression and reduce their negative emotions as much as possible. Hence, their psychological health level is high, and their safety behaviors can be effectively improved.

Campus safety atmosphere reflects the degree of importance attached by a school to individual safety and health within the school and plays a significant role in school safety management. Studies show that a high school safety atmosphere can effectively relieve individual depression and nonconfidence caused by mental stress (16). Therefore, the effect exerted by their mental stress on their RES may rely on the campus safety atmosphere level. When the campus safety atmosphere level is high, students likely turn their mental stress into the motivation to solve the difficulty and to stimulate their confidence in emotion regulation. Thus, the negative influence of mental stress on their RES will be weak. On the contrary, when the campus safety atmosphere level is low, students will probably amplify the negative effect of mental stress. This event will blow their confidence in emotion regulation to a great extent, thereby reducing their RES by a large margin. Hence, the negative effect of mental stress on RES will be great at the time. Campus safety atmosphere level will not only regulate the relationship between mental stress and RES but also may influence the indirect effect of mental stress on PSB through RES.

### Research objects

Before data collection in 2019, ethical approval was obtained from the School Ethics Committees. Moreover, permission was obtained from all participators.

As school bully events considerably take place in middle schools, class teachers and students in three junior high schools in Henan, China were selected as the research objects to collect data. Then, a questionnaire survey at two time points through “class teacher-student” paired survey was given to avoid common method bias. In Phase 1, mental stress and basic individual information questionnaires were given out to students. In Phase 2 (3 months later), RES and campus safety atmosphere questionnaires were distributed, and students’ PSBs were evaluated by class teachers. Before the questionnaire survey, class teachers who would participate in the survey were determined by negotiating with the school, and class teachers randomly selected students in their classes to join in our survey.

In the survey process, the questionnaires of the class teachers could effectively match with those of students in their classes through their job numbers and student numbers. After being filled by the respondents, the questionnaires would be recovered on the spot by our researchers and then numbered. A total of 120 class teachers and 365 middle school students took part in our survey. After invalid questionnaires were excluded, valid paired questionnaires of 112 class teachers and middle school students (an average of 3.12 students for each class teacher) were totally acquired. The numbers of middle school students in grades one, two, and three were 103 (29.4%), 122 (34.9%), and 125 (35.7%), respectively, thereby conforming to the criteria for valid questionnaires. Moreover, the number of male students was 162 (46.3%), whereas that of female students was 188 (53.7%).

### Measuring tool

This study refers to foreign and domestic mature scales. Mental stress of the students was measured through 18 items with large factor loads in the mental stress scale for middle school students (17), including seven dimensions: learning stress, stress from teachers, stress from family environment, stress from parenting style, stress from classmates and friends, social stress, and physiological and psychological stress. RES was measured through a RES scale (18) revised later (19), including three dimensions: self-efficacies regulating positive emotion, depression/pain, and anger/rage, totaling to 12 items. Campus safety was measured via the campus safety atmosphere scale (11) from four aspects: student, administrative department, execution department, and campus environment, including 25 items. PSB was measured through six items (8).

For the adopted foreign mature scales, back-translation procedures were used for translation and back-translation of items were conducted by invited doctoral candidates in English. Moreover, back-translated questionnaires and original questionnaires were compared until no difference is observed. In the questionnaire survey, except for items related to demographic characteristics, Likert five-point scale method was used for the other items. The items were given with 1, 2, 3, 4, and 5 scores according to the following options: totally disagree, disagree, uncertain, agree, and agree very much.

### Statistical method

SPSS 24.0 (Chicago, IL, USA) and AMOS 22.0 were used for data processing and statistical analysis, where statistical methods included the following: confirmatory factor analysis, descriptive statistics and correlation analysis, hierarchy regression analysis, structural equation model, and Bootstrap confidence interval estimation method.

## Results

### Validity and reliability test

Cronbach α values of mental stress, RES, campus safety atmosphere, and PSB scales were 0.937, 0.905, 0.921, and 0.918, respectively. All of these scales were greater than ordinary accepted level 0.80, indicating that the questionnaire was of favorable reliability. Furthermore, AMOS was used to do confirmatory factor analysis of fitting result of structural equation models of the scales (Table 1).

**Table 1:** The result of Confirmatory Factor Analysis

***Variable***	***Factor number***	***χ^2^/df***	***NNFI***	***CFI***	***RMSEA***	***SRMR***
Criterion	–	≤5; ≤3 better	≥0.9	≥0.9	≤0.08	≤0.05
Mental stress	18	1.484	0.960	0.965	0.047	0.039
RES	12	1.675	0.958	0.966	0.056	0.041
Camus safety atmosphere	25	1.267	0.952	0.956	0.035	0.047
PSB	6	2.150	0.980	0.988	0.073	0.026

### Descriptive statistics and correlation analysis

Table 2 shows the descriptive statistics of the main variables. Mental stress presents a significant correlation with RES (*r*=–0.79, *P*<0.01) and PSB (*r*=–0.85, *P*<0.01); RES had a significant positive correlation with PSB (*r*=0.87, *P*<0.01).

**Table 2:** Descriptive statistics and correlation analysis of main variables

***Variable***	***M***	***SD***	***Mental stress***	***RES***	***Campus safety atmosphere***	***PSB***
Mental stress	2.57	0.68	1			
RES	3.08	0.67	−0.79^**^	1		
Campus safety atmosphere	3.04	0.64	−0.80^**^	086^**^	1	
PSB	3.27	0.94	−0.85^**^	0.87^**^	0.87^**^	1

Note:

** represents a significance level of *P*<0.01.

### Direct effect test

**Fig. 2:**
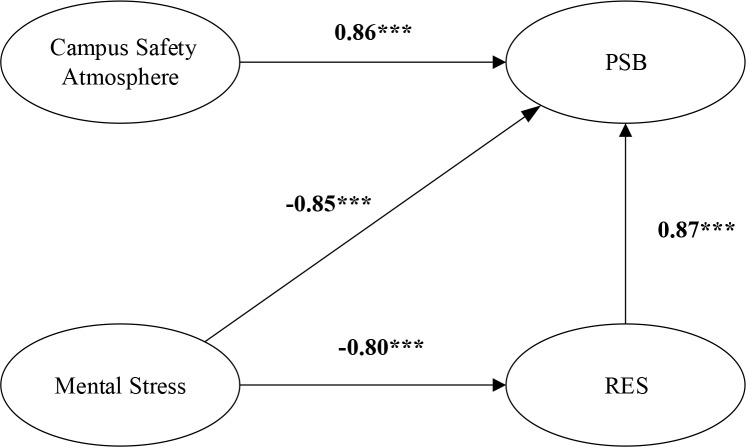
Result of direct effect Note: ^***^ represents a significance level of *P*<0.001

Hierarchical regression models between every two variables were constructed to verify the relationships of mental stress, RES, and campus safety atmosphere with PSB. All direct effect coefficients are summarized in Fig. 2. Mental stress had a significant negative correlation with PSB. Mental stress (*r*=–0.80, *P*<0.001) presented a significant negative correlation with RES. RES (*r*=0.87, *P*<0.001) and campus safety atmosphere (*r*=0. 86, *P*<0.001) presented significant positive correlations with PSB.

### Moderating effect test

**Fig. 3:**
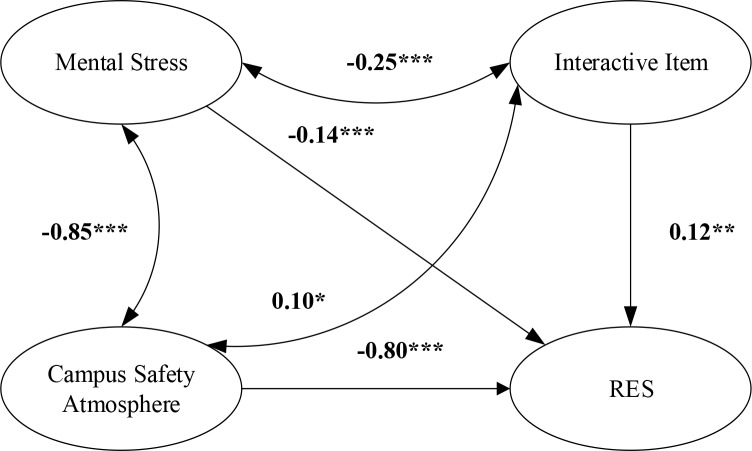
The role of campus safety climate in adjusting mental stress and regulatory emotional self-efficacy Note: ^*^, ^**^, ^***^ represents a significance level of *P*<0.05, *P*<0.01, *P*<0.001, respectively

AMOS was used to verify the moderating effect of campus safety atmosphere between mental stress and RES (Fig. 3). Mental stress could negatively predict RES (*r*=–0.14, *P*<0.001), and campus safety atmosphere could positively predict RES (*r*=0.80, *P*<0.001). In addition, the interactive item between mental stress and campus safety atmosphere was significant (*r*=0.12, *P*<0.01). Thus, the moderating effect of campus safety atmosphere between mental stress and RES was significant.

**Fig. 4:**
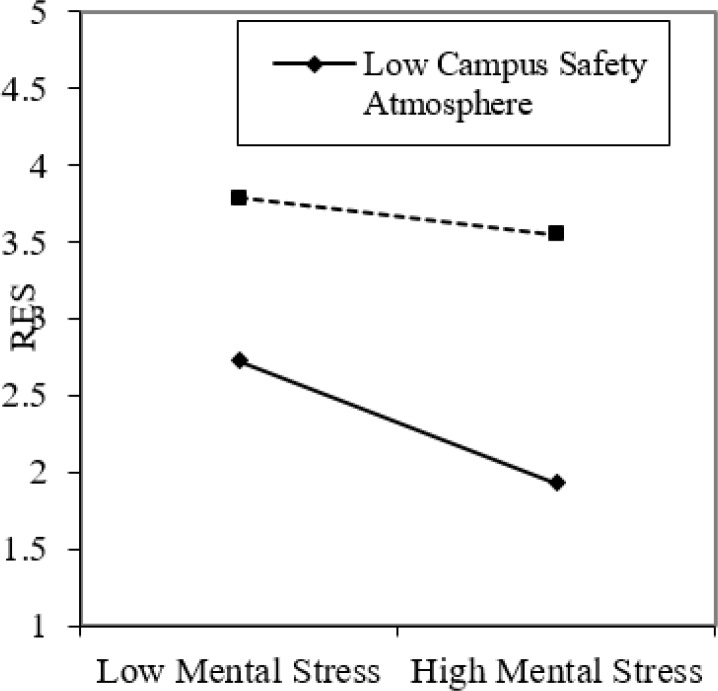
The role of campus safety climate in adjusting mental stress and regulatory emotional self-efficacy

Grouping was conducted according to campus safety atmosphere levels to further confirm whether the moderating effect of campus safety atmosphere between mental stress and RES met the original expectation. The respondents with scores above a standard deviation constituted a group with high campus safety atmosphere score, and those below the standard deviation formed the group with low campus safety atmosphere score. A regression analysis of mental stress for RES was conducted (Fig. 4). It shows that in comparison with low campus safety atmosphere, the negative correlation between mental stress and RES was weak under high campus safety atmosphere.

### Mediating effect and moderated mediating effect test

The Bootstrap method was used to verify the mediating effect and significance of the moderated mediating effect as shown in Table 3. The mediating effect of mental stress on PSB through RES was significant (effect value was −0.57, 95% confidence level was [−0.73, −0.43]).

**Table 3:** The mediating effect and moderated mediating effect test results

***Effect type and adjustment variable***		***Effect value***	***BC 95% CI***
1. Mediating effect: mental stress → RES → PSB		−0.57	[−0.73, −0.43]
2. moderated mediating effect	Low campus safety atmosphere level	−0.25	[−0.37, −0.15]
	Medium campus safety atmosphere level	−0.19	[−0.30, −0.10]
	High campus safety atmosphere level	−0.13	[−0.25, −0.01]
	High–low level difference	0. 12	[0.02, 0.18]

Campus safety atmosphere could moderate the mediating effect of mental stress on PSB via RES. Under low campus safety atmosphere, the indirect effect of mental stress on PSB via RES was significant (indirect effect was −0.25, 95% confidence level was [–0.37, −0.15]). When the campus safety atmosphere was at a medium level, the indirect effect of mental stress on PSB via RES was significant (indirect effect was −0.19, 95% confidence level was [–0.30, −0.10]). When the level was high, the indirect effect was also significant (indirect effect is −0.13, 95% confidence level was [−0.25, −0.01]). As the campus safety atmosphere level rose, indirect effect value would decline.

## Discussion

The results in Fig. 2 showed that the mental stress of middle school students presented a significant negative correlation with their PSB, thus enriching previous theoretical study (20) on the mental stress of middle school students and verifying the harm of mental stress. Therefore, schools and parents should pay high attention to the mental stress of middle school students and jointly take measures to relive their learning, interpersonal, social, and their own physiological and psychological stresses effectively. Specifically, they may pay attention to the psychological health of middle school students from levels of “school-class teacher-parent” and multiple aspects so as to mitigate their mental stress. Moreover, RES of middle school students played a remarkable mediating effect through the negative correlation between mental stress and PSB. Thus, previous research result (15) regarding RES of student group, namely, RES of students played a mediating role among their stress, psychological health, and safety level. In addition, their psychological health and safety level had a direct influence on their safety behaviors, so the perspectives of RES effect were further enriched. Therefore, school administrators should strengthen cultivating students’ RES and actively carry out activities improving their psychological health and confidence. Examples were setting up a psychological counseling and service center and providing necessary psychological guidance. Class teachers should communicate regularly with students to timely understand their learning status, family situation, economic pressure and interpersonal relationship, and timely help, enlighten them, and relieve irreparable harms caused by these stresses.

Moreover, they could help to cultivate their confidence in studying and strengthen their learning abilities. Besides, a harmonious family atmosphere was an important factor influencing the psychological health of middle school students. Hence, parents should set up good examples, show their care for kids, avoid contradictions and conflicts, and create good conditions for their physical and mental development.

The verification results in Fig. 3 and Fig. 4 and Tab. 3 showed that the campus safety atmosphere level exerts an important moderating effect on relieving the mental stress of middle school students and improving their RES and PSB levels. This result was consistent with previous research result (21) regarding the effect of enterprise safety atmosphere. In addition, this finding emphasized the importance of campus safety atmosphere and enriches and improves prior studies on campus safety atmosphere (11). The campus safety atmosphere level could effectively relieve the mental stress of middle school students. Hence, schools should take measures to strengthen the campus safety atmosphere level.

From the above research results, the campus safety atmosphere level should be improved from angles of students, administrative department, campus environment, and execution department. For instance, students could be encouraged to take an active part in campus activities to relieve their mental stress and strengthen their awareness of safety and participation degree in safety. The administrative department should strengthen safety input and formulation of safety standards and effectively improve campus safety level, eating environment, and traffic environment. The execution department should strictly execute various safety rules and regulations and reinforce safety education and safety supervision level. Last, school, teachers, such as class teachers, and parents should cooperate closely to create a good campus, harmonious classes, and safe family atmosphere. The goal was to indirectly mediate their mental stress and reduce the occurrence of safety accidents, such as school violence and bullying.

## Conclusions

Bullies and victims in incidents of school bullying are faced with high mental stress. We found the mental stress of middle school students has a significant negative effect on their PSB. Campus safety atmosphere has a significant positive effect on their PSBs. Moreover, campus safety atmosphere can moderate the relationship between the mental stress of middle school students and their PSB.

## Ethical considerations

Ethical issues (Including plagiarism, Informed Consent, misconduct, data fabrication and/or falsification, double publication and/or submission, redundancy, etc.) have been completely observed by the authors.
